# Synthesis and photocatalytic performance of g-C_3_N_4_/MeTMC-COP composite photocatalyst

**DOI:** 10.3389/fchem.2023.1138789

**Published:** 2023-03-01

**Authors:** Lili Cao, Songli Qiao, Xue Li, Qiang Li

**Affiliations:** College of Science, Heilongjiang Bayi Agricultural University, Daqing, China

**Keywords:** photocatalysis, photodegradation, graphitic carbon nitride, covalent organic polymer, hydrogen production

## Abstract

Covalent organic polymers have excellent application prospects in photocatalysis due to their excellent visible light absorption and structural designability. However, their fast recombination efficiency and complex preparation process limit their applications. Because of the above problems, this paper used urea to prepare g-C_3_N_4_ by high-temperature thermal polymerization and prepared g-C_3_N_4_ composite photocatalyst loaded with MeTMC-COP (g-C_3_N_4_/MeTMC-COP) by hydrothermal method. The photocatalytic hydrogen generation and photocatalytic degradation capabilities of composite photocatalysts with various mass ratios were investigated by characterizing the catalyst and using the organic dye Rhodamine B (RhB) as the pollutant. According to the research, the specific surface area of the g-C_3_N_4_/MeTMC-COP composite may reach 40.95 m^2^ g^−1^ when the mass ratio of g-C_3_N_4_ and MeTMC-COP is 3:1 (25.22 m^2^ g^−1^). It can offer more active sites for the photocatalytic process, and because the fluorescence peak intensity is the lowest, it has the lowest photogenerated electron-hole recombination efficiency. In comparison to g-C_3_N_4_, 3:1 g-C_3_N_4_/MeTMC-COP can breakdown rhodamine B up to 100% after 75 min of light irradiation; its photocatalytic hydrogen generation efficiency is 1.62 times that of g-C_3_N_4_, and the hydrogen evolution rate is 11.8 μmol g^−1^ h^−1^.

## Introduction

Since the beginning of the 21st century, with the rapid development of modern industry, human beings have been committed to scientific and technological progress, leading to an increasingly severe energy crisis and environmental pollution. It is estimated that the global energy demand will double to the present by 2050 (Islam et al., 2018). At present, the development of most industries still relies on fossil fuels such as coal, oil, and natural gas. The extensive use of such fossil fuels will lead to the emission of harmful gases such as SO_2_ and NO, which will pollute the environment (Hosseini and Wahid, 2016; Wu et al., 2017), and they are not renewable. Therefore, the development of green energy is significant.

Hydrogen is a secondary clean energy with a high heat of combustion. The product is only water and has no pollution to the environment. It is considered to be an ideal substitute for fossil energy. Therefore, hydrogen can be used to change the current energy usage and reduce the use of fossil energy. Traditional industries use coal gasification, methane reforming, and petroleum cracking to produce hydrogen, which consumes many fossil fuels and causes environmental pollution. However, hydrogen production by electrolysis of water has a great demand for electric energy and high cost, which could be more conducive to sustainable development. Biological hydrogen production uses microorganisms to decompose, photolyze, and ferment biomass. It is an environmentally friendly hydrogen production method. However, it requires high pressure and temperature, which could be more conducive to large-scale hydrogen production (Akhlaghi and Najafpour-Darzi, 2020). Solar energy has become the most popular research direction because of its inexhaustible, green, and pollution-free characteristics (Chen et al., 2019). Many scientists are currently focusing on studying how solar photocatalytic semiconductors may produce hydrogen energy (Shen et al., 2020; Li et al., 2019; Sun et al., 2021).

Fresh water is one of the necessities for human survival. Currently, 2.53% of the total water is fresh on land. Among them, 68.69% cannot be exploited, and only 0.3% of the water can meet human beings’ daily production and living needs. The world produces an average of about 4 billion tons of wastewater every year, some of which will be degraded by the self-purification system of the water body, and a large part will cause the deterioration of the entire ecosystem (Lu et al., 2015), thereby causing a shortage of freshwater resources. Although many policies on water body protection have been issued, the increase in population and social development has led to increasing global demand for fresh water. Wastewater treatment is an inevitable and urgently needed solution for my country’s development. Dyes, widely used in printing and dyeing, textile, and other fields, are common organic pollutants in water pollution. About 700,000 tons of dyes are produced a year, of which about 15,000 tons are discharged into the water, discoloring the water body and reducing the self-purification capacity of the water system. Plants can also not perform photosynthesis because sunlight is difficult to irradiate, and some dye wastewater is carcinogenic and teratogenic (Rafatullah et al., 2010; Cheng and Tsai, 2017).

Graphitic carbon nitride (g-C_3_N_4_) is a novel kind of green photocatalyst popular in various industries due to its inexpensive cost of raw materials, straightforward manufacturing method, and non-toxic qualities (Ran et al., 2021; Wang et al., 2018) (Muhammad et al., 2020). The ultraviolet-visible light absorption band edge position of (g-C_3_N_4_ is 460 nm (Patnaik, Martha, and Parida 2016), the absorption utilization rate of visible light is low, and the recombination efficiency of photogenerated carriers is fast, which limits its application field. Numerous efficient methods have been developed to modify g-C_3_N_4_, including noble metal deposition (Patnaik, Sahoo, and Parida, 2018), element doping (Yi et al., 2020), surface morphology modulation (Han et al., 2015) (Zhu et al., 2022), construction of heterojunctions (Song et al., 2020), and others, in order to increase the visible light response of g-C_3_N_4_ and decrease the recombination efficiency of photogenerated electron-hole pairs (Shi et al., 2022; Wu et al., 2022). Na and Fe co-doped g-C_3_N_4_ composites were produced by (Wang, He, and Nan 2021b) and used in the photocatalytic degradation of methylene blue (MB) dyes. The impact of photocatalytic degradation varies according to the Fe and Na content. MB’s adsorption capacity and degradation rate rose from 1.16% to 56.7%, and 89.6%–98.4%, respectively, when some of the Fe was substituted by Na. By creating a heterojunction of three-dimensional Fe_2_O_3_ and two-dimensional g-C_3_N_4_, (Wang et al., 2021a), improved the responsiveness of the catalyst to visible light and the electrical conductivity and encouraged the separation of photogenerated electron-hole pairs. Within 10 min, 8%Fe_2_O_3_/g-C_3_N_4_ photocatalytic degradation effect on amanita reached 97.6%.

Metal elements and metal oxides are often employed to produce heterojunction modifications and the doping of g-C_3_N_4_. In this study, graphitic carbon nitride composites (g-C_3_N_4_/MeTMC-COP) were made by hydrothermally preparing g-C_3_N_4_ by thermal condensation, then using dimethyl sulfoxide and ethanol as solvents. MeTMC-COP loading is aided by - interactions produced by the - conjugated system in the g-C_3_N_4_ and MeTMC-COP structures. Additionally, the development of an amide bond between the carboxyl group in the MeTMC-COP structure and the amino group in g-C_3_N_4_ might react to create a more substantial contact between the two. Finally, the photocatalytic degradation of rhodamine B and the photocatalytic hydrogen generation performance of the composite catalyst were investigated. Its shape, structure, and photoelectric performance were examined using several characterizations.

## Materials and methods

### Materials

The urea supplier (99% purity) was Shanghai Siyu Chemical Technology Co., Ltd. Shanghai Macklin Biochemical Technology Co., Ltd. Supplied the rhodamine B. The Lizhi Primary Battery Sales Department, Yingze District, Taiyuan City, provides polyvinylidene fluoride (D210C). Tianjin Damao Chemical Reagent Factory supplied the N, N-dimethylformamide. Tianjin Fuyu Fine Chemical Co., Ltd. Supplied the dimethyl sulfoxide used in this study. The Tianjin Damao Chemical Reagent Factory gave us pure ethanol. The supplier of triethanolamine was Tianjin Kemiou Chemical Reagent Co., Ltd. Analytical grade chemicals are used throughout.

### Preparation of g-C_3_N_4_/MeTMC-COP

**FIGURE 1:**
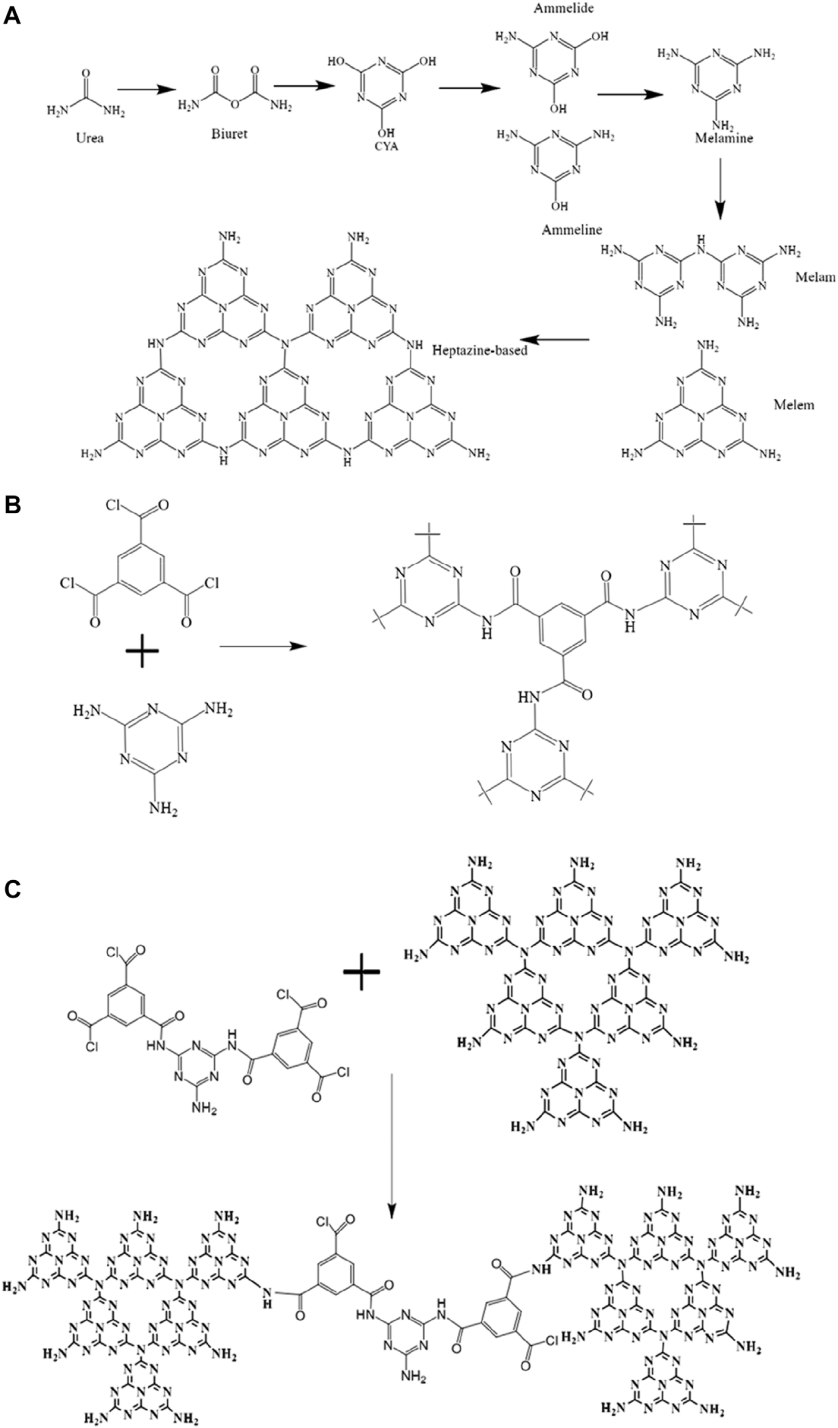
**(A)** The synthetic process for g-C_3_N_4_; **(B)** MeTMC-COP; **(C)** a schematic showing how g-C_3_N_4_/MeTMC-COP is made.

g-C_3_N_4_: Put 20 g of urea in a ceramic crucible that holds 50 mL and has a cover. Placing the ceramic crucible in the muffle furnace, bringing the temperature up to 30°C initially, raising it to 520°C at a rate of 3°C/min, and holding it there for 2.5 h. Pale yellow g-C_3_N_4_ powder was produced after cooling in the furnace (the reaction equation is shown in Figure 1A).

MeTMC-COP: Weigh trimesoyl chloride and melamine monomers with a molar ratio of 1:1, and quickly grind them evenly. The solid powder was added into the reactor, and the reaction temperature was set at 200°C. After reacting for 14 h, the obtained yellow blocky solid product was ground into powder. After washing with acetone, deionized water was Soxhlet extracted for 2 days. Finally, it was dried under vacuum at 80°C (the synthetic route is shown in Figure 1B).

g-C_3_N_4_/MeTMC-COP: g-C_3_N_4_ was loaded with MeTMC-COP using a straightforward solvothermal technique. Weigh 0.18 g of g-C_3_N_4_ and 0.018 g of MeTMC-COP (10:1) and add them to 20 mL of a DMSO and 100% ethanol solution (1:1). For 12 h, stir magnetically to equally distribute the two. Place the mixture in the reactor, turn the heat up to 150°C, and let it sit for 12 h to react. Suction-filter the blended mixture. The dimethyl sulfoxide solution and deionized water were used to wash the solid powder two to three times. A composite catalyst made of 10:1 g-C_3_N_4_/MeTMC-COP was produced by vacuum drying at 70°C. The aforesaid methods were followed in the development of the 3:1 g-C_3_N_4_/MeTMC-COP and 5:1 g-C_3_N_4_/MeTMC-COP composite catalysts (the schematic diagram of the preparation is shown in Figure 1C).

### Characterization

Instruments: muffle furnace (SG-XL1200, Shanghai Institute of Optics and Fine Mechanics); scanning electron microscope (Quanta 400F, Philips); transmission electron microscope (JEM-2010, JEOL Ltd.); fluorescence spectrophotometer (LS55, United States PE company); X-ray diffractometer (XRD-600, Shimadzu, Japan); Fourier transform infrared spectrometer (VERTEX70, Bruker, Germany); UV-visible spectrophotometer (solid) (UV-2600, Shimadzu, Japan); Ultraviolet-visible spectrophotometer (liquid) (UV-2550, Shimadzu, Japan); xenon lamp light source (CEL-HXF300, Beijing Zhongjiao Jinyuan Technology Co., Ltd.); specific surface area tester (NOVA3200e, Quanta Corporation, United States).

### Electrochemical performance test

The equipment utilized in the electrochemical lab is a CHI660E type (Shanghai Chenhua). The working electrode is an ITO glass covered with a sample (12 cm^2^, the sample area is 11 cm^2^), and the reference electrode is a Hg/HgCl_2_/KCl electrode. The counter electrode is a platinum sheet (11 cm^2^). The electrolyte is an aqueous solution of 0.5 M Na_2_SO_4_. In a typical three-electrode cell, electrochemical tests were carried out.

### Performance test of photocatalytic degradation of organic pollutants

The visible light source is a 300 W xenon lamp with UVIRCUT420 ultraviolet cutoff filter and Vis-REF visible light reflection filter (through 420–780 nm visible light). The produced materials’ photocatalytic activity was assessed using RhB as the target pollutant.

To make the catalyst equally disseminated, weigh 50 mg of the catalyst and add it to the RhB solution with a volume of 50 mL and a concentration of 0.01 mol/mL. After reaching the adsorption equilibrium, the xenon lamp was turned on, and 2.5 mL samples were obtained at the same intervals under the condition of light. Stirring was maintained for 1 h under the condition of avoiding light. By using a microporous filter membrane, the photocatalyst was removed. The filtrate was then collected, and the absorbance at the wavelength of maximum absorption, 464 nm, was determined. According to the Lambert-Beer law (Rafatullah et al., 2010), using the standard working curve of RhB, the actual concentration of RhB in the filtrate under different illumination times was calculated, and then the photocatalytic degradation rate of RhB was calculated.

### Performance test of photocatalytic hydrogen production from water

The light source simulation was a 300 W xenon lamp with filter glass, and cooling water was pumped to keep the solution temperature below 25°C. In order to completely mix the ingredients, first combine 20 mL of deionized water, 5 mL of triethanolamine, and 10 mg of catalyst in a beaker. The uniformly mixed solution is placed in a photolysis water hydrogen production reactor, and a visible light source is placed above the reactor. Vacuumize the photocatalytic water hydrogen production reaction system until the vacuum degree of the system drops to 3 kPa. Make sure that the air in the device is completely exhausted, turn on the xenon lamp, and catalyze hydrogen production from water with light. The concentration of hydrogen was detected by gas chromatograph every half hour.

## Results and discussion

### Structural performance characterization

#### Chemical components

**FIGURE 2:**
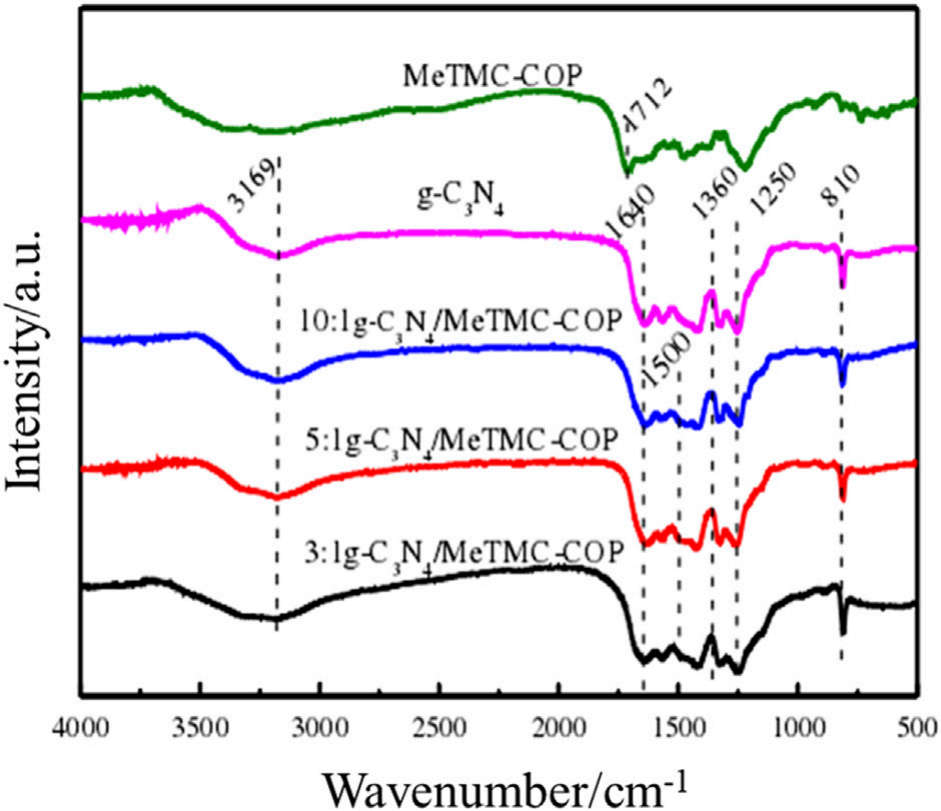
FT-IR spectra of different ratios of g-C_3_N_4_/Me TMC-COP.

The infrared spectrum of composite catalysts with various compositions is shown in Figure 2. The stretching vibration of carboxylic acid may be seen in the image as the distinctive MeTMC-COP infrared peak at 1712 cm^−1^. The triazine unit’s distinctive plane bending vibration peak may be found in the absorption peak of g-C_3_N_4_ at 810 cm^−1^. The stretching characteristic peaks of the C-N heterocycles in the structure are many peaks within the range of 1,250–1,640 cm^−1^ (Bajiri et al., 2021; Yang et al., 2021). The broad absorption peak observed at around 3,169 cm^−1^ is the stretching vibration of -NH and NH_2_. It shows that g-C_3_N_4_ was successfully prepared from urea by thermal condensation, and the terminal amino group still exists in the material (Dong et al., 2013). This provides a prerequisite for the connection of the composite photocatalyst g-C_3_N_4_/MeTMC-COP through chemical bonds. After loading MeTMC-COP, the infrared characteristic peaks and C-N and C=N peaks of the triazine structure still exist. A very weak infrared characteristic peak of amide bond appears at 1,500 cm^−1^. The peak at 1,640 cm^−1^ weakened after loading, further confirming the reaction between COP and g-C_3_N_4_. The change of the infrared characteristic peaks is not obvious, which is due to the less amount of MeTMC-COP in the composite and the stronger stretching vibration of g-C_3_N_4_ (Kumar et al., 2019).

### Crystal structure

**FIGURE 3:**
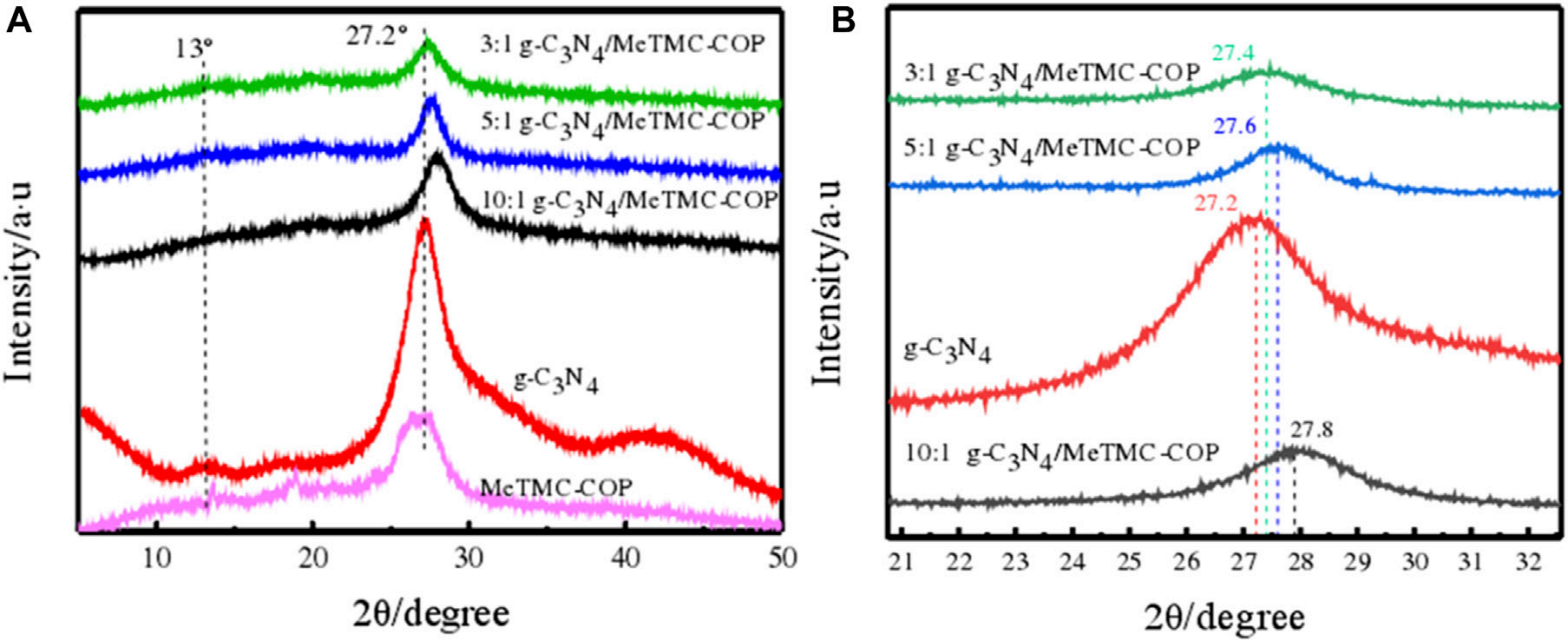
XRD patterns **(A)** and partial enlarged images **(B)** of different ratios of g-C_3_N_4_/MeTMC-COP.

XRD patterns were used to further evaluate the g-C_3_N_4_/MeTMC-COP composite material’s crystallinity. The crystallinity of g-C_3_N_4_, MeTMC-COP, and g-C_3_N_4_/MeTMC-COP with various mass ratios is depicted in Figures 3A, B, respectively. XRD spectra and related, magnified local pictures. The figure shows that g-C_3_N_4_/MeTMC-COP has prominent diffraction peaks at 27.2° for a variety of mass ratios. The weaker diffraction peak at 13° corresponds to the (100) crystal plane, with an interplanar distance of d = 0.327 nm (Liang et al., 2017) and corresponding to the (002) crystal plane. According to reports in the literature (Sreekanth et al., 2017), these two diffraction peaks can be linked to stacking between the molecular planes of the aromatic rings and the periodic arrangement of the triazine ring structure. After doping MeTMC-COP, the composite material’s diffraction peaks resemble those of g-C_3_N_4_ and lack a distinctive MeTMC-COP peak. This may be caused by the low MeTMC-COP concentration and poor crystallinity of the composite material, which also demonstrates that MeTMC-COP doping has little impact on the packing structure and crystallinity of g-C_3_N_4_.

The peak at 27.2° changes after adding MeTMC-COP compared to pure g-C_3_N_4_, as can be seen from the partial magnification of Figure 3B. As the doping ratio varies, the degree of the two shift increases. This could be because MeTMC-COP alters the lattice spacing by introducing defects into the g-C_3_N_4_ crystal. As g-C_3_N_4_ is doped with MeTMC-COP at this location, the interlayer accumulation of g-C_3_N_4_ is inhibited, causing the strength of the diffraction peak to drop (Shan et al., 2016).

### Microscopic morphology analysis

**FIGURE 4:**
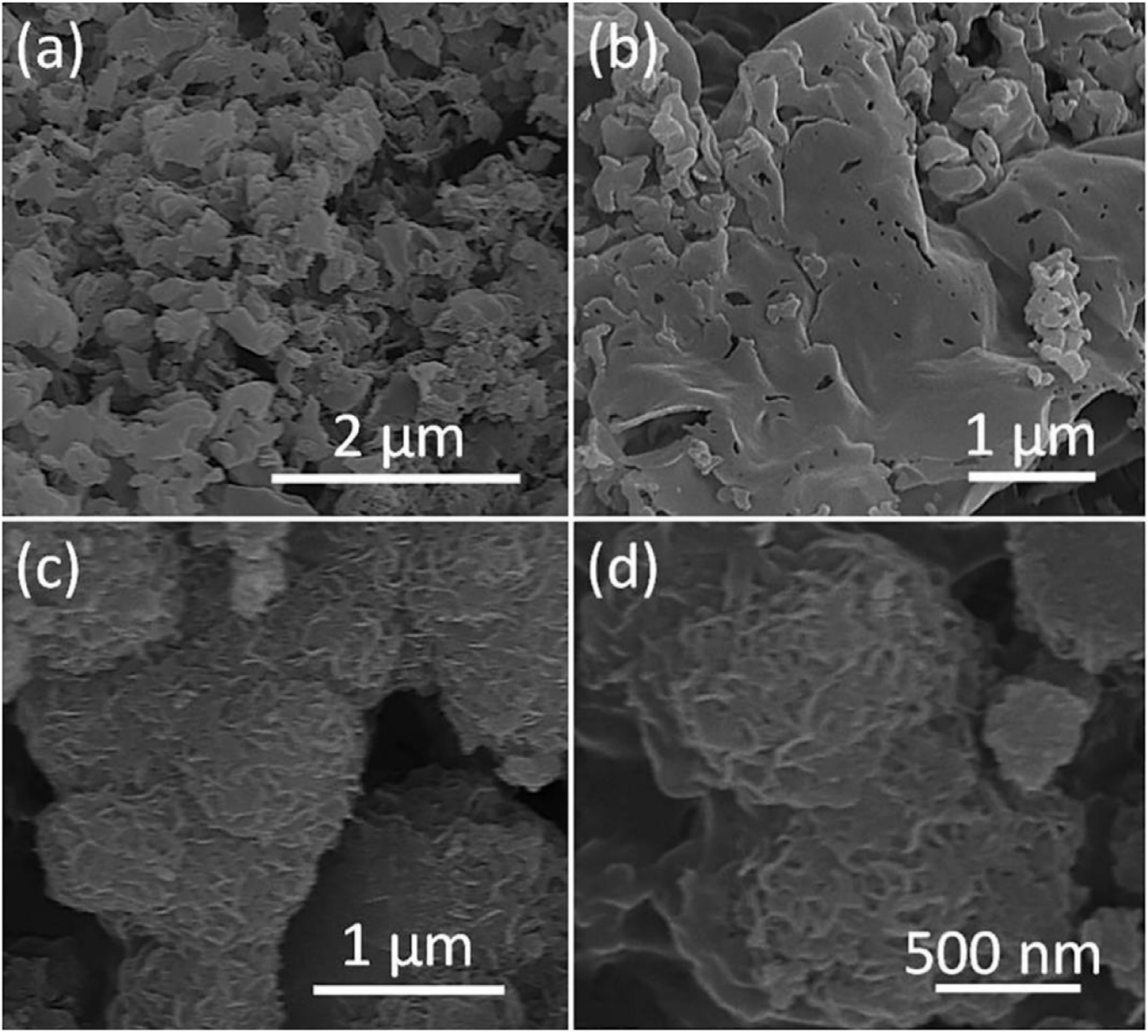
SEM images of g-C_3_N_4_ **(A)** MeTMC-COP **(B)** 3:1 g-C_3_N_4_/MeTMC-COP **(C)** 5:1 g-C_3_N_4_/MeTMC-COP **(D)**.

The microscopic morphology of the composite photocatalyst g-C_3_N_4_/MeTMC-COP also influences its photocatalytic performance. The SEM images of g-C_3_N_4_/MeTMC-COP with various doping ratios are shown in Figure 4. It is evident from the SEM picture of g-C_3_N_4_ in Figure 4A that g-C_3_N_4_ has a smooth, graphite-like sheet-like shape. This is because when urea is selected as the precursor to prepare g-C_3_N_4_, urea undergoes thermal decomposition during high-temperature thermal polymerization and forms a sheet structure through molecular self-assembly. In addition, the π-π interaction between g-C_3_N_4_ molecules makes smaller lamellar structures stack each other to form a graphite-like smooth lamellar micromorphology (Thomas et al., 2008). After doping the lamellar MeTMC-COP, the microscopic morphology of the composite photocatalyst did not change significantly, and the lamellar stacking still occurred. It shows that the three-dimensional morphology of g-C3N4 does not change with the doping of MeTMC-CO. With the doping of MeTMC-COP, part of the channel structure appeared in the irregular lamellar structure, the microscopic morphology of the material became loose, and the lamellar smoothness decreased. This is because MeTMC-COP and g-C_3_N_4_ are disseminated in the solution during the hydrothermal preparation process, bringing the two polymers into complete contact. Through Physical contacts and chemical bonding, MeTMC-COP is evenly distributed throughout the surface of g-C_3_N_4_, increasing the number of active sites for the photocatalytic reaction and enhancing the catalytic activity of the composite photocatalyst.

### Specific surface area and pore size distribution

**FIGURE 5:**
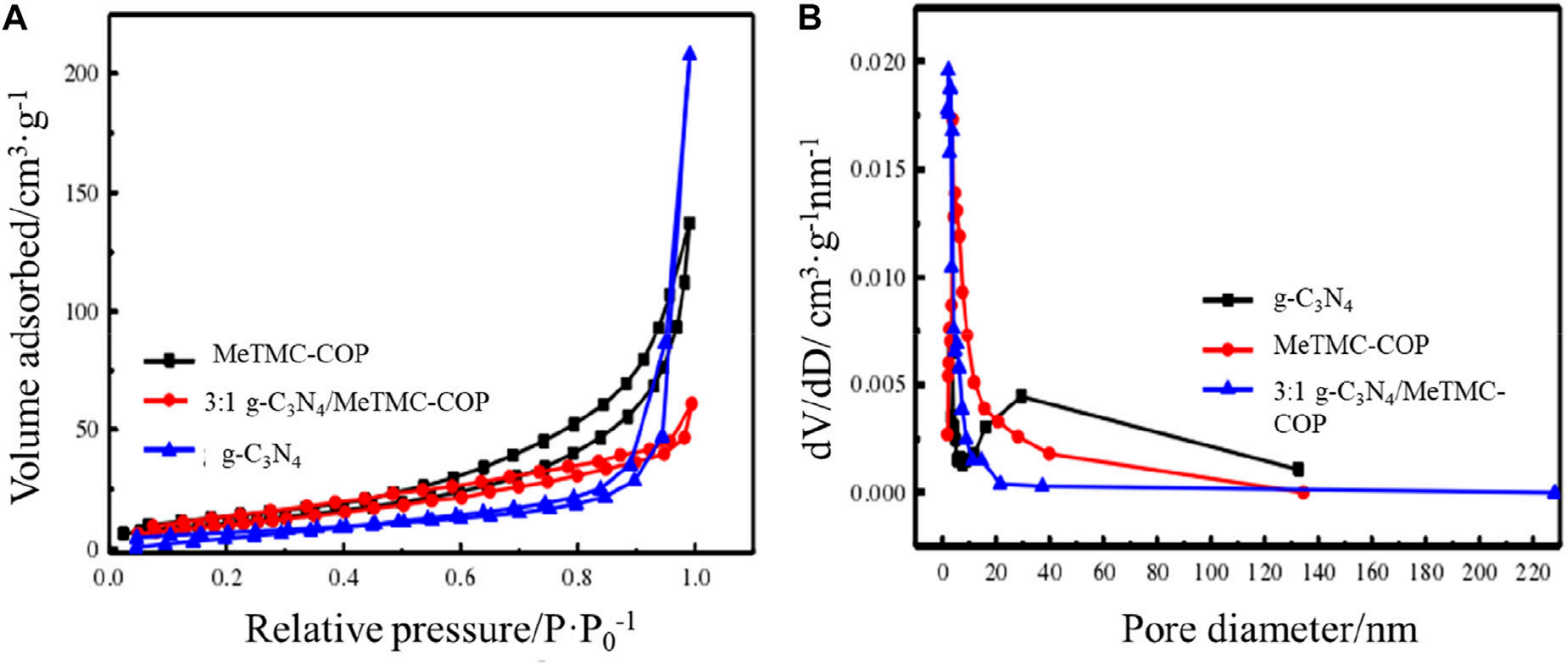
Nitrogen adsorption-desorption isotherm **(A)** and pore size distribution diagram **(B)** of g-C_3_N_4_, 3:1 g-C_3_N_4_/MeTMC-COP, MeTMC-COP.

The photocatalytic performance of photocatalysis is related to its active sites, and the number of active sites is related to its specific surface area. By conducting nitrogen adsorption-desorption studies at 77K, the specific surface area and pore size distribution of the composite photocatalyst g-C_3_N_4_/MeTMC-COP were investigated and determined. The nitrogen adsorption-desorption isotherm of MeTMC-COP, g-C_3_N_4_, and 3:1 g-C_3_N_4_/MeTMC-COP is shown in Figure 5A, and the catalyst’s pore size distribution map is shown in Figure 5B. The nitrogen adsorption-desorption isotherms of g-C_3_N_4_, 3:1 g-C_3_N_4_/MeTMC-COP, and MeTMC-COP may be inferred from the relative pressure (P/P_0_) hysteresis loop’s curve shape in Figure 5A. Designated as type IV. The specific surface area of g-C_3_N_4_ has been calculated to be 25.22 m^2^ g^−1^. The specific surface area of the modified g-C_3_N_4_/MeTMC-COP composite increases to 40.95 m^2^ g^−1^ after doping MeTMC-COP. It could be because the two catalysts are connected by chemical bonds, which lessens the degree of g-C_3_N_4_ polymerization. It demonstrates that the catalyst’s specific surface area is impacted by the addition of MeTMC-COP. According to (Lin et al., 2018), there are more photocatalytically active sites, which promotes photocatalysis. After doping, the composite material’s specific surface area (42.81 m^2^ g^−1^) is somewhat less than that of the MeTMC-COP material, although the difference is not very great. It could be because MeTMC-COP has a lot of g-C_3_N_4_ loaded on its surface as a result of a - interaction.

The Barrett–Joyner–Halenda (BJH) technique was used to examine and compute the catalyst’s pore size distribution. Figure 5B demonstrates the vast variety of pore sizes present in the three catalysts (2–140 nm). MeTMC-COP pores are mostly spread at 10 nm in a 3:1 g-C_3_N_4_/MeTMC-COP mixture, whereas graphitic carbon nitride pores are primarily concentrated around 30 nm. The pore size distribution can be used to determine if the catalysts are mesoporous materials.

### Photoelectrochemical analysis

**FIGURE 6:**
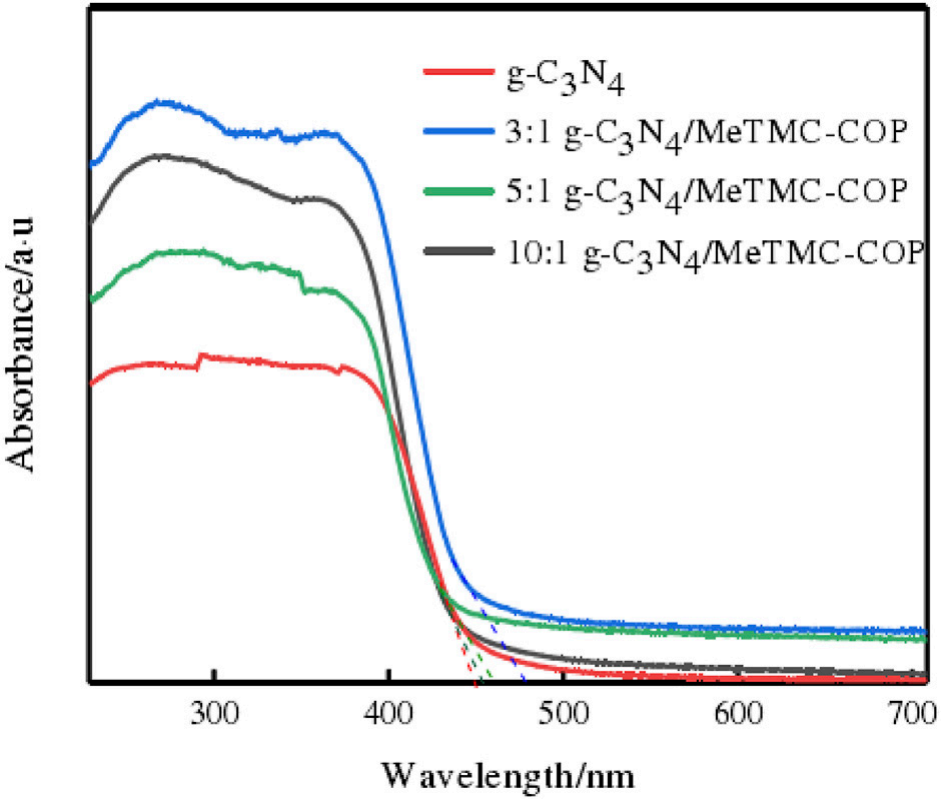
UV-Vis diffuse emission spectra of g-C_3_N_4_ and different ratios of g-C_3_N_4_/MeTMC-COP.

The photocatalytic performance of the catalyst is significantly influenced by its band structure and photoresponsiveness. By using UV-Vis diffuse reflectance spectroscopy, the absorption edge and photoresponse intensity of g-C_3_N_4_/MeTMC-COP were examined. The UV-Vis diffuse reflectance pictures of g-C_3_N_4_ and g-C_3_N_4_/MeTMC-COP with various ratios are shown in Figure 6. At 450 nm, g-C_3_N_4_ has an absorption sideband that may be seen. As a result, it can be deduced that g-C_3_N_4_ has a bandgap energy of 2.75 eV and effectively absorbs light with a wavelength of 450 nm or less.

When the catalyst was doped with MeTMC-COP, the absorption intensity of the composite catalyst to light (blue-violet, *λ* < 456 nm) increased, and the responsiveness to visible light also increased. The catalyst’s absorption sideband is moved from 450 nm to 472 nm in the red. This indicates that following modification, the composite material’s visible light absorption is enhanced, as well as its photoresponsiveness, which is favorable to the excitation and creation of photogenerated electrons and holes at lower energy, and its visible light consumption rate. It has been discovered that the restricted band width is the narrowest when the ratio of the two is 3:1. The carrier’s use of visible light is currently at its maximum and the energy needed to excite it is at its lowest.

It is clear from the aforementioned observation and discussion that the highly conjugated structure of the MeTMC-COP material, which is included in the g-C_3_N_4_/MeTMC-COP composite photocatalyst, enhances the electron delocalization effect. In addition, a heterostructure was created when MeTMC-COP and g-C_3_N_4_ combined.

**FIGURE 7:**
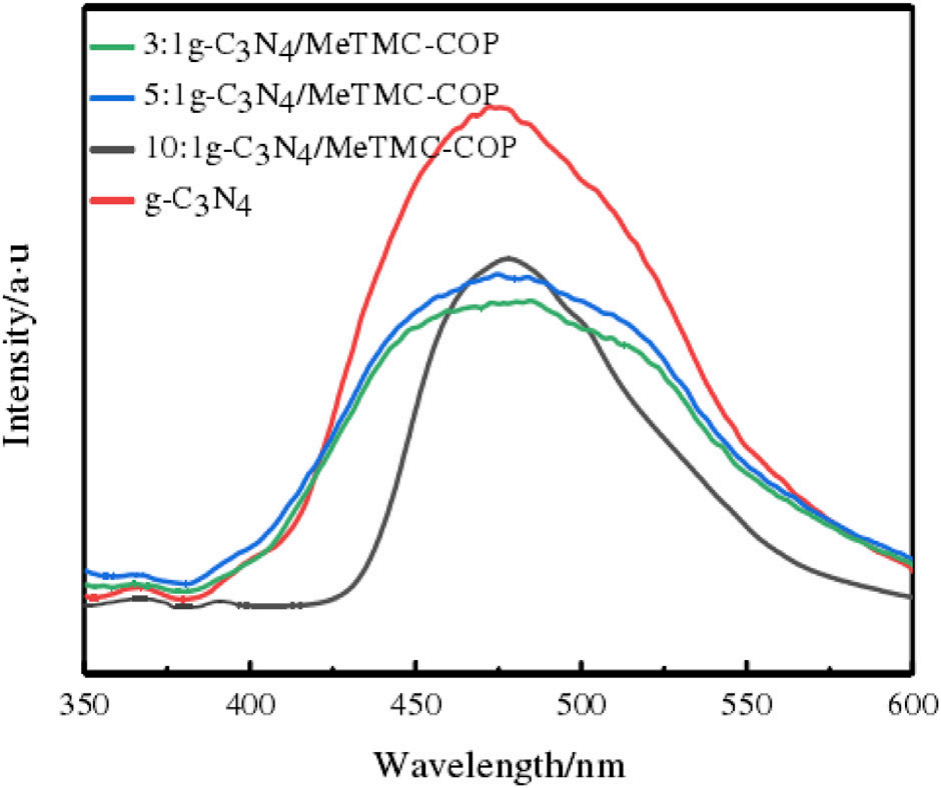
PL spectra of g-C_3_N_4_ and g-C_3_N_4_/MeTMC-COP at different ratios.

Intensity of photoluminescence (PL) spectrum was used to examine the transfer, separation, and recombination of photogenerated carriers in order to investigate the impact of adding MeTMC-COP on the photocatalytic activity of g-C_3_N_4_. Figure 7 displays the PL spectra of g-C_3_N_4_ and various g-C_3_N_4_/MeTMC-COP ratios. The fluorescence peak intensity of g-C_3_N_4_ is considerably greater when compared to composite materials. This suggests that the photocatalytic process cannot take place because g-C_3_N_4_ photogenerated electron-hole recombination may occur more quickly. The composite’s fluorescence intensity dropped when MeTMC-COP was doped. This is due to high conjugated structure will partially prevent carriers from recombining, as seen by the lowest PL intensity in the 3:1 g-C_3_N_4_/MeTMC-COP sample. This suggests that the photogenerated carriers can currently be quickly separated and transferred. Additionally, because of the low recombination rate of photogenerated carriers at this ratio, more carriers can migrate to the surface of the composite photocatalyst. In order to fulfill the purpose of photocatalysis, more active molecules are produced and more target molecules participate in redox processes. The formation of the photogenerated electron-hole recombination center, which hinders carrier migration, occurs gradually as the doping level changes.

**FIGURE 8:**
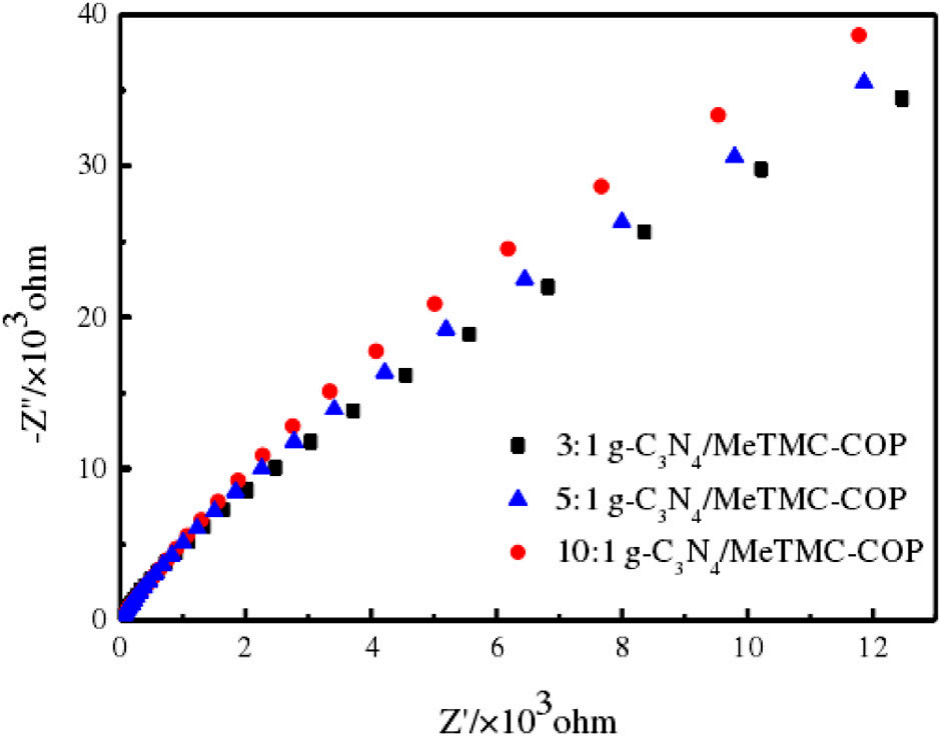
EIS spectra of different ratios of g-C_3_N_4_/MeTMC-COP.

The internal resistance of the charge transfer process in the sample may be measured by electrochemical AC impedance. In general, the width of the semicircle arc of electrochemical impedance is inversely proportional to the electron transfer resistance value. The photocatalytic process is more favorable with a smaller diameter because it allows for greater separation and transmission of photogenerated electrons and holes as well as reduced electron transfer resistance. On the other hand, charge separation becomes more challenging the bigger the diameter (Chang and Park, 2010). The catalyst sample Nyquist plot is shown in Figure 8. As the quantity of doping varies, the radius of the composite material likewise varies, as seen in Figure 8. The heterostructure modified the energy level structure of the composite from 3:1 g-C_3_N_4_/MeTMC-COP to 5:1 g-C_3_N_4_/MeTMC-COP to 10:1 g-C_3_N_4_/MeTMC-COP, which increased the electron delocalization impact. This shows that the catalyst experiences reduced resistance to light irradiation, the charge transfer rate is quicker, and the photocatalytic effect is at its greatest when the ratio of g-C_3_N_4_/MeTMC-COP is 3:1.

### Photocatalytic performance

**FIGURE 9:**
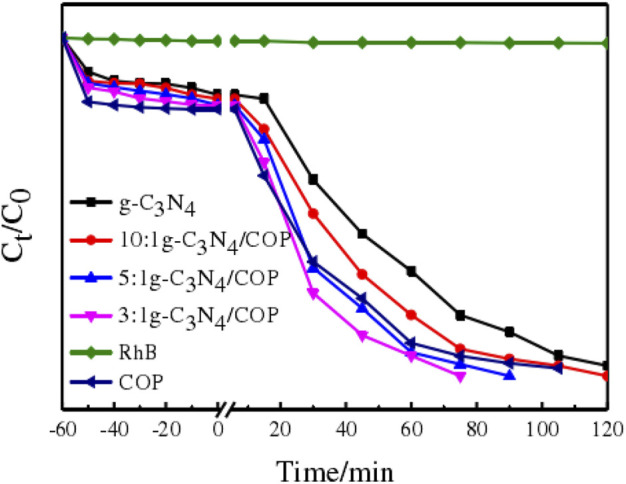
Degradation-time curves of RhB degradation by different ratios of g-C_3_N_4_/MeTMC-COP.

As visible light, a 300 W xenon lamp (>420 nm) was selected. To investigate the photocatalytic activity of the samples, Rhodamine B was chosen as the target pollutant, and photocatalytic performance was assessed. The catalytic efficiency of the photocatalytic degradation of the dye rhodamine B by g-C_3_N_4_/MeTMC-COP composites in various ratios is depicted in Figure 9. The figure shows that the catalyst in the reaction system attained an adsorption-desorption equilibrium for the RhB dye solution after 60 min of dark reaction. Subsequently, the xenon lamp was turned on for photocatalytic degradation, and samples (2.5 mL) were taken every 15 min. Within the first 15 min of illumination, the dye solution added with the catalyst showed obvious photocatalytic effect. The photocatalytic efficiency of the pure rhodamine B solution without catalyst did not change significantly. Degradation rate of rhodamine B solution by g-C_3_N_4_, MeTMC-COP 3:1 g-C_3_N_4_/MeTMC-COP, 5:1 g-C_3_N_4_/MeTMC-COP, 10:1 g-C_3_N_4_/MeTMC-COP after 75 min of light irradiation reached 82%, 94%, 100%, 96.5%, 92%, respectively. It is discovered that, when MeTMC-COP is added as a dopant, the composite material’s catalytic action is enhanced when compared to pure g-C3N4. On the one hand, the composite material’s increased specific surface area offers more photocatalytic active sites. On the other hand, the transfer efficiency of photogenerated carriers is increased as a result of the creation of conjugated structures and heterojunctions. But when the composition changes, the photocatalytic activity is hampered by the shadowing effect. The material’s better photocatalytic degradation effect after doping MeTMC-COP is made possible by its higher responsiveness to visible light when combined with the characterization analysis mentioned above. Reduced recombination rate and greater specific surface area of photogenerated electrons.

**FIGURE 10:**
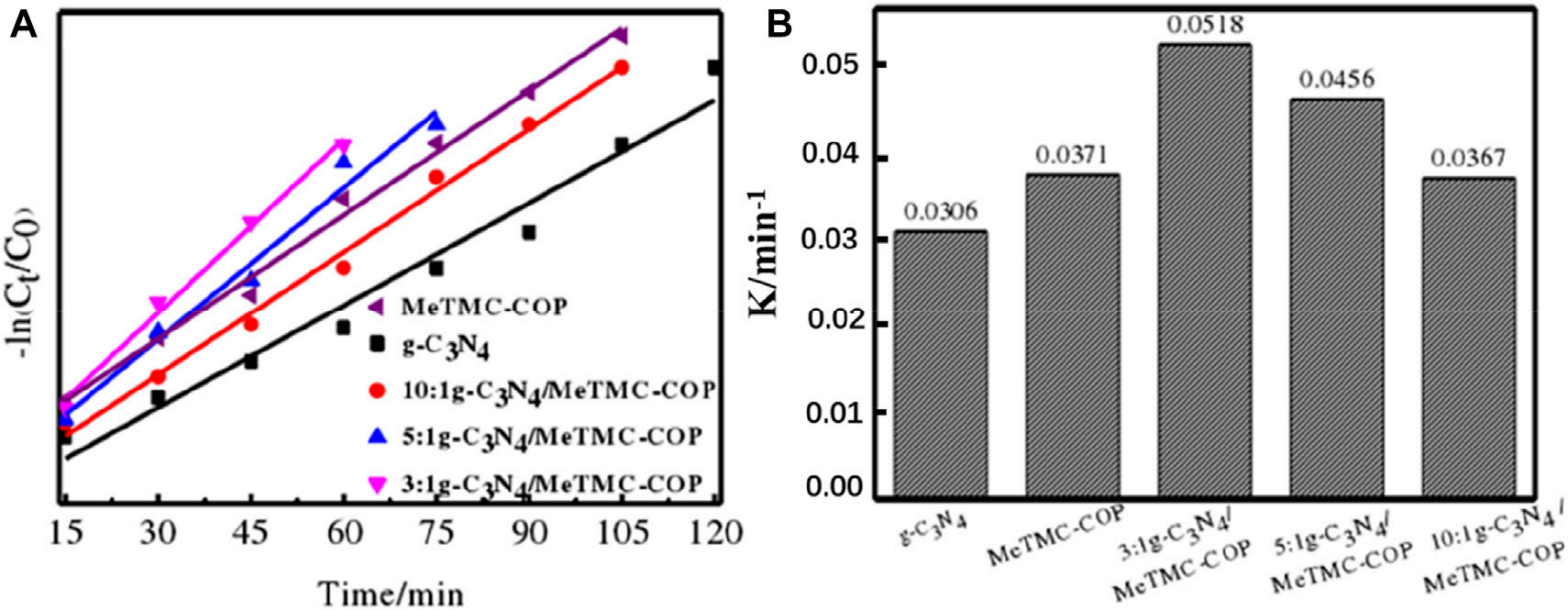
Photocatalytic kinetic curves **(A)** and reaction rate constants **(B)** of g-C_3_N_4_/MeTMC-COP with different proportions.

Figure 10 depicts the kinetic fitting curve for the photocatalytic degradation of RhB. The samples g-C_3_N_4_, MeTMC-COP, 3:1 g-C_3_N_4_/MeTMC-COP, 5:1 g-C_3_N_4_/MeTMC-COP, and 10:1 g-C_3_N_4_/MeTMC-COP had linear fitting correlation coefficients (*R* ^2^) of 0.96659, 0.9932, 0.99178, 0.97417, and 0.9909, respectively. It demonstrates that the RhB degradation by the catalyst under visible light may be fitted by the first-order reaction kinetic equation. According to Figure 10B, the reaction rate constants (k) for the catalysts g-C_3_N_4_, MeTMC-COP, 3:1 g-C_3_N_4_/MeTMC-COP, 5:1 g-C_3_N_4_/MeTMC-COP, and 10:1 g-C_3_N_4_/MeTMC-COP are 0.0305 min^-1^, 0.0371 min^-1^, 0.0518 min^-1^, 0.0456 min^-1^, and 0.0367 min^-1^, respectively.

**FIGURE 11:**
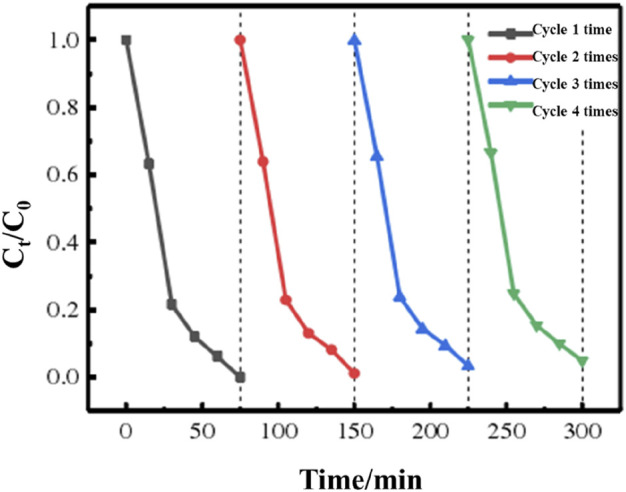
Cyclic stability graph of 3:1 g-C_3_N_4_/MeTMC-COP degraded RhB under visible light.

For photocatalysts to be used in real manufacturing and in real life, stability is also essential. To assess the materials’ photocatalytic stability and effectiveness, the organic dye cycle experiment was conducted under simulated visible light. The cycle stability diagram of the 3:1 g-C_3_N_4_/MeTMC-COP deteriorated RhB solution is shown in Figure 11 when seen in visible light. The figure shows that the degrading impact did not dramatically alter when the catalyst was regenerated four times. RhB can still degrade at a rate of over 95% despite a very little reduction of photocatalytic activity. This demonstrates that the 3:1 g-C_3_N_4_/MeTMCCOP composite may be used to produce items for modern civilization and has consistent performance and an excellent reaction to visible light.

**FIGURE 12:**
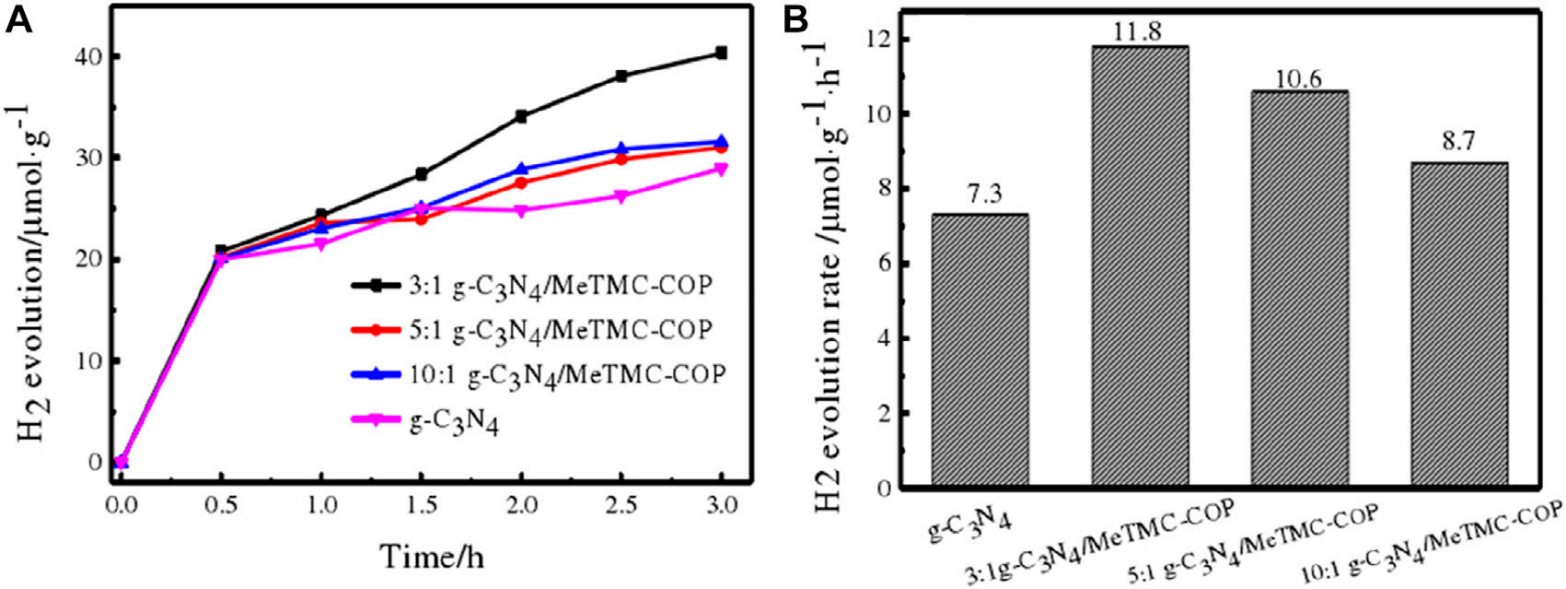
Time curves for photocatalytic hydrogen generation using g-C_3_N_4_ are shown in **(A)**, along with rate constants**(B)** for various g-C_3_N_4_/Me TMC-COP ratios.

Following the foregoing description, the composite photocatalyst exhibits outstanding photocatalytic action, as demonstrated by EIS, UV-Vis DRS, PL, and BET. After investigating the photocatalytic destruction of organic contaminants on a sample, it was used in the creation of photocatalytic hydrogen. Triethanolamine was employed as a sacrifice substance, a xenon light (>420 nm) lamp was chosen to replicate the visible light source, and the experimental variables were regulated to ensure consistency. Figure 12 displays the composite catalyst’s rate of hydrogen generation. The ratio of the hydrogen production rate of composite materials with various ratios is shown in the figure to be as follows: 3:1 g-C_3_N_4_/MeTMC-COP>5:1 g-C_3_N_4_/MeTMC-COP>10:1 g-C_3_N_4_/MeTMC-COP > g-C_3_N_4_. In comparison to pure g-C_3_N_4_, the composite’s ability to produce hydrogen after doping was enhanced. This is due to the heterojunction that is created between the two photocatalysts once the semiconductor material has been doped, which speeds up the migration of the composite material’s photogenerated carriers. This heterojunction is created through chemical bonding and intermolecular interactions. Consequently, the bandgap width is lowered and electron-hole recombination is inhibited. Triethanolamine also serves as an electron donor sacrificial agent, consuming holes and lowering the recombination of electron-hole pairs produced by photosynthesis. Additionally, it may successfully stop the composite photocatalyst from getting photocorroded while catalyzing the synthesis of hydrogen from water. According to Figure 12B, the photocatalytic hydrogen generation rate constants for 3:1 g-C_3_N_4_/MeTMC-COP, 5:1 g-C_3_N_4_/MeTMC-COP, 10:1 g-C_3_N_4_/MeTMC-COP, and g-C_3_N_4_ are 11.8, 10.6, 8.7, and 7.3 μmol g^-1^·h^−1^, respectively. The composite performs best at 1.62 g-C_3_N_4_ of photocatalytic water hydrogen generation when the ratio is 3:1.

## Conclusion

This work proposes and synthesizes a novel class of covalent organic polymer based on amide bond coupling with the aim of addressing the present issues of water pollution and energy deficit. A hydrothermal approach was used to create the g-C_3_N_4_/MeTMC-COP composite photocatalyst, and the effects of various ratios on the composite’s performance were investigated. According to the findings, the 3:1 g-C_3_N_4_/MeTMC-COP exhibited the shortest band gap, smallest radius, and lowest fluorescence intensity of any EIS map. Its specific surface area rose to 40.95 m^2^ g^−1^ in comparison to g-C_3_N_4_. It indicates that MeTMC-COP loading improves the catalyst’s number of photocatalytic active sites, which is advantageous to the photocatalytic process. Rhodamine B may degrade at a rate of 100% after 75 min of exposure to visible light, which is 2.36 times faster than pure g-C_3_N_4_. Additionally, the greatest photocatalytic hydrogen generation effect is achieved by 3:1 g-C_3_N_4_/Me TMC-COP, which has a rate constant of 11.80 μmol g^−1^ h^-1^, or around 1.62 times that of g-C_3_N_4_.

## Data Availability

The original contributions presented in the study are included in the article/supplementary material, further inquiries can be directed to the corresponding author.
